# [Retracted] Astragaloside IV ameliorates high glucose-induced HK-2 cell apoptosis and oxidative stress by regulating the Nrf2/ARE signaling pathway

**DOI:** 10.3892/etm.2025.12979

**Published:** 2025-09-22

**Authors:** Jing Wang, Hong-Min Guo

Exp Ther Med 17:4409–4416, 2019; DOI: 10.3892/etm.2019.7495

Following the publication of this paper, it was drawn to the Editor's attention by a concerned reader that flow cytometric assay (FCM) data featured in Fig. 2B on p. 4412 were strikingly similar to FCM data which were ultimately published in other papers in different journals that were written by different authors at different research institutes, one of which was submitted to the journal *Biomedicine & Pharmacotherapy* and has since been retracted.

Owing to the fact that the contentious data in the above article were found to be strikingly similar to data that had already been submitted for publication elsewhere in other papers in different journals, the Editor of *Experimental and Therapeutic Medicine* has decided that this paper should be retracted from the Journal. The authors were asked for an explanation to account for these concerns, but the Editorial Office did not receive a satisfactory reply. The Editor apologizes to the readership for any inconvenience caused.

